# Primary focal segmental glomerulosclerosis in a patient with ankylosing spondylitis: A rare presentation requiring a broad differential in nephrotic syndrome

**DOI:** 10.1002/ccr3.8901

**Published:** 2024-05-14

**Authors:** Pooya Zardoost, Sana Tyabuddin, Austin Cantu, Musa Abu‐Jubara, Jackson Mittlesteadt, Henry Wehrum

**Affiliations:** ^1^ OhioHealth Doctors Hospital Columbus Ohio USA; ^2^ Ohio University Heritage College of Osteopathic Medicine Dublin Ohio USA

**Keywords:** acute medicine, chronic diseases, general medicine, nephrology, rheumatology

## Abstract

Ankylosing spondylitis (AS) presents with renal failure and proteinuria in a minority of cases, usually due to secondary amyloidosis or IgA nephropathy. While focal segmental glomerulosclerosis (FSGS) is less common, it should still be in the differential regardless of the patient's clinical profile.

## 1 INTRODUCTION

Ankylosing spondylitis (AS), characterized by a chronic inflammatory disease of the axial skeleton, sacroiliac, and peripheral joints, has been reported to also affect extra‐articular organs.^1^ Renal involvement has been noted in a minority of cases, most of which are due to secondary amyloidosis.^1^, ^2^ Focal segmental glomerulosclerosis (FSGS) is a less common etiology in this patient group but is nevertheless a leading and growing cause of kidney disease worldwide.^2^, ^3^ We present a case from our institution of a patient with a history of AS who was admitted for hypertensive emergency and nephrotic range proteinuria. Renal biopsy was remarkable for FSGS. In addition, the FSGS subtype was consistent with a primary subtype, despite her history of obesity and obstructive sleep apnea (OSA) raising suspicion for secondary FSGS. As the prevalence of FSGS is increasing worldwide, providers should have a higher suspicion for this disease, regardless of history.

## 2 CASE REPORT

A woman in her early thirties with a history significant for AS, OSA, and recently diagnosed hypertension (2‐month history) presented after a hypertensive reading of 230/155 mmHg at her optometry appointment. She denied any symptoms of fever, headache, dizziness, chest pain, vision changes, chest pain, or shortness of breath. Her home medications were hydrochlorothiazide 25 mg daily, sulfasalazine 1000 mg twice a day, and adalimumab 40 mg subcutaneous injection every 14 days.

**FIGURE 1:**
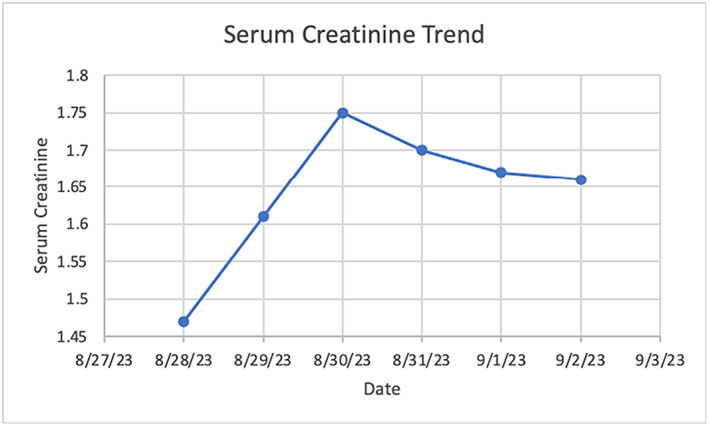
Patient's serum creatinine trend throughout hospitalization. Note the peak of serum creatinine on the second day of hospitalization, this coincided with improvement of blood pressure on the diltiazem infusion.

On admission she was hypertensive at 201/133 mmHg, tachycardic at 107 beats per minute, and saturating 99% on room air. Aside from 2+ pitting edema of the lower extremities, the physical exam was unremarkable. Cardiopulmonary examination was non‐revealing as confirmed on radiography, electrocardiogram only showed sinus tachycardia. Computed tomography (CT) of the head was negative for acute intracranial pathology. Creatinine was 1.47 mg/dL as shown on Figure 1, with her baseline at 0.7 mg/dL, suggesting acute kidney injury (AKI).

## 3 METHODS

She received several doses of intravenous hydralazine, and after lack of improvement was started on a diltiazem infusion, which was chosen due to persistent tachycardia. Renal ultrasound revealed symmetric normal sized kidneys with preserved cortical thickness and no hydronephrosis. Serum albumin was 1.8 g/dL and remained below 2 g/dL throughout the 6 days of admission. Urinalysis revealed hazy clarity, with specific gravity of 1.028, proteinuria, and glucosuria above our laboratory's upper limit as greater than 500 mg/dL, with small blood noted with a red blood cell count of 5 per high‐power field. A protein:creatinine ratio was ordered, which revealed a ratio outside of our laboratory's range of measurements, with urine protein reported as above 600 mg/dL and urine creatinine at 114.0 mg/dL, suggesting a ratio of at least above 6 g. An extensive secondary hypertension workup as well as autoimmune investigation consisting of ANA, anti‐scleroderma antibody, paraproteinemia workup, C3, and C4 was non‐revealing. A 24‐hour urine protein study resulted in 11.30 g in 24 h.

**FIGURE 2:**
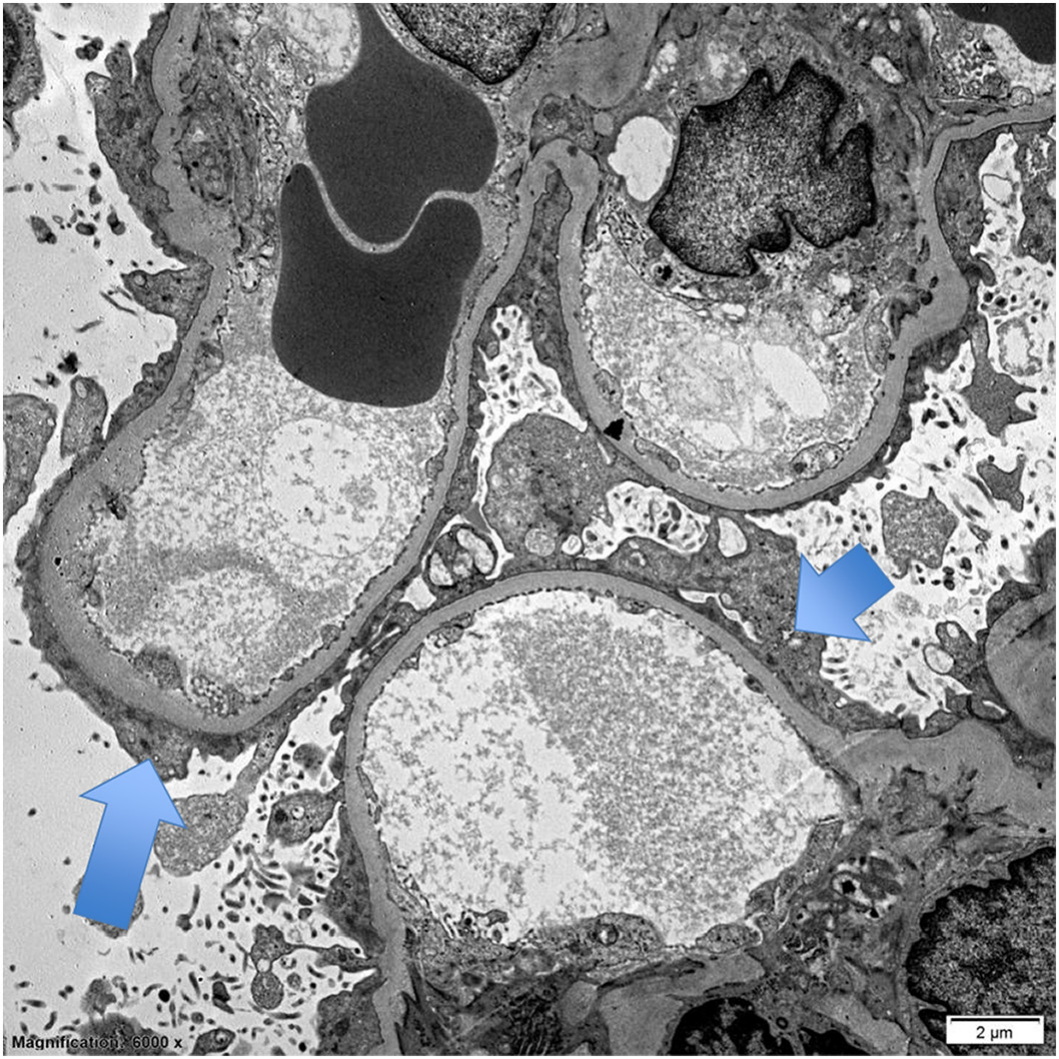
Electron microscopy of patient's glomerular filtration barrier. Emphasized is a section with complete podocyte foot effacement (blue arrows), with normal glomerular basement membrane thickness, and no electron dense deposits.

**FIGURE 3:**
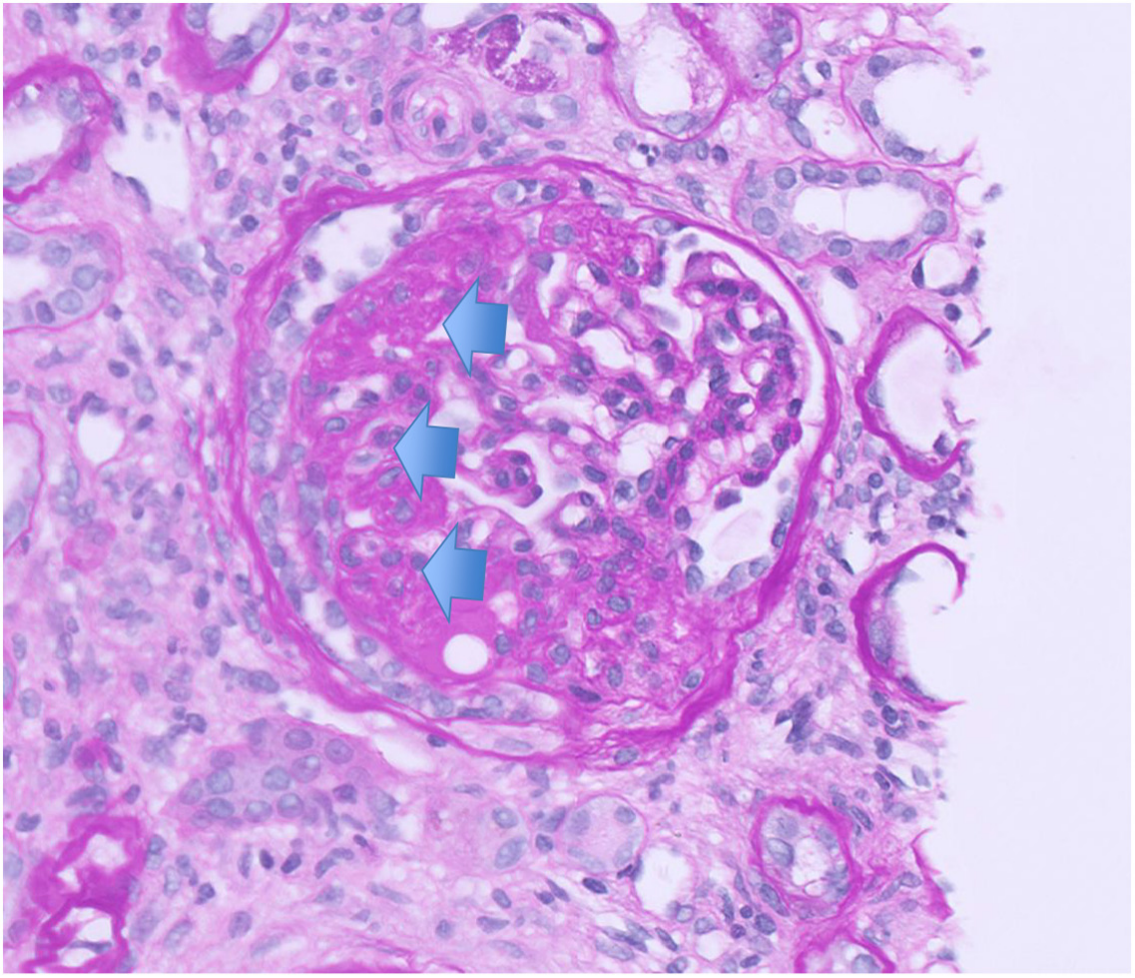
Periodic acid–Schiff (PAS) stain of a glomerulus from renal biopsy. Emphasized is the section of segmental sclerosis (blue arrows).

The patient's blood pressure lowered throughout admission and her tachycardia resolved. She was transitioned from the diltiazem infusion to labetalol 400 mg three times daily, amlodipine 10 mg daily, and hydrochlorothiazide at 50 mg daily. Due to lack of improvement in serum creatinine levels, she underwent a kidney biopsy which returned significant for complete podocyte process effacement on electron microscopy, normal glomerular basement membrane thickness, and no electron dense deposits consistent with primary FSGS (Figure 2). Histology was also remarkable for findings consistent with FSGS (Figure 3).

## 4 CONCLUSION AND RESULTS

The patient was discharged from our facility with the same antihypertensive regimen she was transitioned to during admission. She was scheduled for a referral to see a nephrologist outpatient for her diagnosis of FSGS and to initiate steroid treatment.

## 5 DISCUSSION

This case presented many intriguing findings. Her clinical profile falls into the minority of cases of AS presenting with renal disease. In addition, this specific population usually presents with nephrotic syndrome that is due to secondary amyloidosis and IgA nephropathy. This patient's renal biopsy returned diagnostic of FSGS. Despite her history of obesity and OSA leading one to suspect a diagnosis of adaptive FSGS, the electron microscopy demonstrated primary FSGS.

Our patient's clinical triad of hypoalbuminemia, edema, and persistent proteinuria of greater than 3.5 g/24 h were diagnostic of nephrotic syndrome.^4^ Persistent proteinuria can be the result of glomerular dysfunction, tubulointerstitial disease, secretory proteinuria, or overflow proteinuria.^5^ Normal autoimmune and complement studies made a glomerular cause less likely. The level of bands in the serum protein electrophoresis also did not explain a predominant overflow etiology to the level of protein excretion observed, which would have been more common in amyloidosis.^5^ The patient's serum creatinine peaked on the second day of hospitalization and steadily decreased, which coincided with improvement in blood pressure control with diltiazem. This is due to renal autoregulation as the patient's afferent arterioles became less constricted as the blood pressure improved.^6^ However, the lack of improvement to the patient's baseline creatinine in the setting of nephrotic range proteinuria warranted biopsy.

Kidney disease in AS is rare but is associated with increased mortality in this population.^2^ Most common etiologies are amyloidosis, followed by IgA nephropathy.^2^ Autoimmunity can cause a systemic disturbance of immunity with the central feature being loss of tolerance to normal cellular and/or extracellular proteins, pertaining to many sites including the glomerulus.^7^ FSGS has rarely been reported in this population.

FSGS is often postulated to be multifactorial in etiology, but the common consequence is podocyte injury.^3^ As podocytes have an inability to replicate, loss of podocytes leads to hypertrophy of remaining podocytes to cover more of the glomerular capillary surface.^3^ FSGS has several subtypes including primary and secondary.^8^ Secondary causes consist of adaptive, genetic, virus, and medication‐associated.^3^ Adaptive FSGS is associated with hyperfiltration states, such as obesity, OSA, or high‐protein diet.^3^ Our patient had a BMI of 43.2 and a history of OSA, which raised suspicion for adaptive FSGS. However, renal biopsy reveals only partial podocyte effacement, whereas our patient had complete effacement consistent with primary FSGS. Our patient did not receive genetic testing, and it is plausible that she had genetic variants that played a role in her podocyte injury.

Various medications have been associated with FSGS including bisphosphonates, lithium, sirolimus, and anthracycline medications including doxorubicin and daunomycin.^3^ Our patient's medications consisted of hydrochlorothiazide and sulfasalazine. There are no known cases, to our knowledge, of FSGS related to hydrochlorothiazide or adalimumab. There have been case reports of FSGS related to sulfasalazine, but this was not continued during admission and her creatinine continued to rise and her proteinuria persisted, making this medication less likely to be the culprit.^9^

Primary FSGS, formerly termed idiopathic FSGS, has been associated with circulating cytokines causing podocyte foot process effacement.^10^, ^11^ Primary FSGS is defined by ruling out the likelihood of other forms of FSGS and is presumed to be caused by a yet unidentified podocyte‐toxic factor and is often amenable to treatment.^11^ The complete effacement of the foot processes of the podocytes on microscopy, as demonstrated on Figure 2, as well as proteinuria greater than 3.5 g/day and albumin consistently below 3 g/dL confirmed the diagnosis of primary FSGS in our patient. Unlike secondary subtypes, primary FSGS is expected to respond to glucocorticoid or immunosuppressive therapy. Treatment consists of high‐dose glucocorticoid therapy with prednisone at a single dose of 1 mg/kg for a maximum of 80 mg for at least 4 weeks and then until complete remission is achieved or 16 weeks, followed by a taper.^8^, ^11^ Remission is determined by reduction in proteinuria, with complete remission defined as a reduction of proteinuria to less than 0.3 g/day, or less than 300 mg/g urine creatinine and normal serum creatinine and serum albumin.^8^, ^11^ Second‐line agents in the setting of persistent proteinuria include cyclosporine or mycophenolate.^11^ Renin–angiotensin–aldosterone system (RAAS) blockade is recommended as well.^8^

Primary FSGS is a leading cause of kidney disease across patients of all clinical profiles. As research into FSGS continues to grow, providers should have a lower threshold to investigate for this cause of proteinuria, even when history suggests another cause. Providers placing primary FSGS higher in the differential diagnoses will help improve outcomes, considering that if diagnosis is confirmed via biopsy, it can be successfully treated with steroids.

## AUTHOR CONTRIBUTIONS

**Pooya Zardoost:** Conceptualization; investigation; project administration; writing – original draft; writing – review and editing. **Sana Tyabuddin:** Writing – original draft. **Austin Cantu:** Data curation; investigation. **Musa Abu‐Jubara:** Investigation. **Jackson Mittlesteadt:** Data curation. **Henry Wehrum:** Supervision; writing – review and editing.

## CONFLICT OF INTEREST STATEMENT

The authors have no conflicts of interest to disclose.

## CONSENT

Written informed consent was obtained from the patient to publish this report in accordance with the journal's patient consent policy.

## Data Availability

Data sharing not applicable as no new data were generated.

## References

[1] Kim TJ , Kim TH . Clinical spectrum of ankylosing spondylitis in Korea. Joint Bone Spine. 2010;77(3):235‐240. doi:10.1016/j.jbspin.2009.11.015 20356776

[2] Akdoğan MF , Gücün M , Denizli N , et al. Complete reversal of nephrotic syndrome secondary to amyloidosis with use of infliximab in a patient with inflammatory bowel disease and ankylosing spondylitis. Ren Fail. 2011;33(5):531‐533. doi:10.3109/0886022X.2011.577543 21574898

[3] Rosenberg AZ , Kopp JB . Focal segmental Glomerulosclerosis [published correction appears in Clin J Am Soc Nephrol. 2018 Dec 7;13(12):1889]. Clin J Am Soc Nephrol. 2017;12(3):502‐517. doi:10.2215/CJN.05960616 28242845 PMC5338705

[4] McCloskey O , Maxwell AP . Diagnosis and management of nephrotic syndrome. Practitioner. 2017;261(1801):11‐15. 29020719

[5] Haider MZ , Aslam A . Proteinuria. StatPearls. Treasure Island (FL); 2023. https://www.ncbi.nlm.nih.gov/books/NBK564390/

[6] Palmer BF , Clegg DJ . Renal considerations in the treatment of hypertension. Am J Hypertens. 2018;31(4):394‐401. doi:10.1093/ajh/hpy013 29373638

[7] Gorenjak M . 4. Kidneys and autoimmune disease. EJIFCC. 2009;20:28‐32. 27683324 PMC4975267

[8] Chapter 6: idiopathic focal segmental glomerulosclerosis in adults. Kidney Int Suppl. 2011;2(2):181‐185. doi:10.1038/kisup.2012.19 PMC4089762 25018931

[9] Abdallah E , Al‐Helal B , Asad R , Kannan S , Draz W . Focal segmental glomerulosclerosis secondary to Mesalamine use in a patient with ulcerative colitis. SM Journal of Nephrology and Therapeutics. 2017;2:1‐4. doi:10.36876/smjnt.1008

[10] Guruswamy Sangameswaran KD , Hashmi MF , Baradhi KM . Focal segmental glomerulosclerosis. StatPearls. StatPearls Publishing; 2023 https://www.ncbi.nlm.nih.gov/books/NBK532272/30335305

[11] Rovin BH , Adler SG , Barratt J , et al. Executive summary of the KDIGO 2021 guideline for the Management of Glomerular Diseases. Kidney Int. 2021;100(4):753‐779. doi:10.1016/j.kint.2021.05.015 34556300

